# Large Isoforms of UNC-89 (Obscurin) Are Required for Muscle Cell Architecture and Optimal Calcium Release in *Caenorhabditis elegans*

**DOI:** 10.1371/journal.pone.0040182

**Published:** 2012-07-02

**Authors:** Patrick M. Spooner, Jennifer Bonner, Andres V. Maricq, Guy M. Benian, Kenneth R. Norman

**Affiliations:** 1 Center for Cell Biology and Cancer Research, Albany Medical College, Albany, New York, United States of America; 2 Department of Pathology, Emory University, Atlanta, Georgia, United States of America; 3 Department of Biology, Skidmore College, Saratoga Springs, New York, United States of America; 4 Department of Biology, University of Utah, Salt Lake City, Utah, United States of America; University of Pennsylvannia, United States of America

## Abstract

Calcium, a ubiquitous intracellular signaling molecule, controls a diverse array of cellular processes. Consequently, cells have developed strategies to modulate the shape of calcium signals in space and time. The force generating machinery in muscle is regulated by the influx and efflux of calcium ions into the muscle cytoplasm. In order for efficient and effective muscle contraction to occur, calcium needs to be rapidly, accurately and reliably regulated. The mechanisms underlying this highly regulated process are not fully understood. Here, we show that the *Caenorhabditis elegans* homolog of the giant muscle protein obscurin, UNC-89, is required for normal muscle cell architecture. The large immunoglobulin domain-rich isoforms of UNC-89 are critical for sarcomere and sarcoplasmic reticulum organization. Furthermore, we have found evidence that this structural organization is crucial for excitation-contraction coupling in the body wall muscle, through the coordination of calcium signaling. Thus, our data implicates UNC-89 in maintaining muscle cell architecture and that this precise organization is essential for optimal calcium mobilization and efficient and effective muscle contraction.

## Introduction

Muscle contraction is triggered by the rapid entry of calcium, a ubiquitous and diverse signaling molecule, into the muscle cytosol. Thus, cytosolic levels of calcium need to be reliably and accurately regulated both spatially and temporally for effective muscle contraction. The sacromere contains several giant proteins, including titin, nebulin and obscurin, which are hypothesized to be critical components involved in sarcomere assembly and organization. While both titin and nebulin have been postulated to be molecular scaffolds that have roles in dictating the length of myofilaments [1], [2], the role of obscurin, the most recently discovered giant muscle protein, in sarcomerogenesis and sarcomere function is not well understood. Obscurin is a member of the twitchin/titin branch of the immunoglobulin (Ig) superfamily and is a modular protein composed of several adhesion and signaling domains [2]. Obscurin predominantly localizes to the M-line where it participates in myofilament assembly and organization [3]–[8]. However, a recent study has shown that mice homozygous for an obscurin deletion allele display normal sarcomeric organization [9]. Yet, a small obscurin isoform containing a tandem kinase domain (known as KIAA1639) [10], [11], was still expressed in the obscurin knock out mice [9]. In addition, a close homolog of obscurin, obscurin-like 1 (obsl1), which only contains Ig domains, still localized normally to the M-line [9]. These data suggest that obsl1 and the small tandem kinase isoform of obscurin might compensate for the absence of full length obscurin.

In addition to its localization to the M-line, obscurin associates with the sarcoplasmic reticulum (SR), a key storage organelle for calcium. This association is mediated by a direct interaction of obscurin with the SR membrane protein small Ankyrin 1 [9], [12], [13]. Disruption of obscurin by small interfering RNAs or knock out of the large obscurin isoform leads to disorganization of the SR [6], [7], [9]. The molecular and physiological consequences of this disorganization are not known.

Unlike mammals, the *C. elegans* genome contains only one obscurin homolog, UNC-89, which like obscurin localizes to the M-line and has been implicated in myofilament assembly and organization [14]–[16]. Similar to mammalian obscurin, UNC-89 is a giant multi-domain protein consisting primarily of many Ig domains, two fibronectin type 3 domains, a Src homology 3 (SH3) domain, a Dbl homology (DH) domain, a plekstrin homology (PH) domain, and two protein kinase domains [17]. In this study, we have found that UNC-89 is required for normal adult sacromere and SR organization. Furthermore, we have found that mutants lacking the large Ig domain-rich isoforms of UNC-89 have reduced calcium signaling and reduced muscle activity. Thus, we propose that UNC-89 is vital for maintaining muscle cell cytoarchitecture, which is crucial for optimal calcium mobilization and excitation-contraction (E-C) coupling in *C. elegans* muscle.

## Results

### unc-89 mutations suppress the physiological consequences of calcium dysregulation in muscle

**Figure 1:**
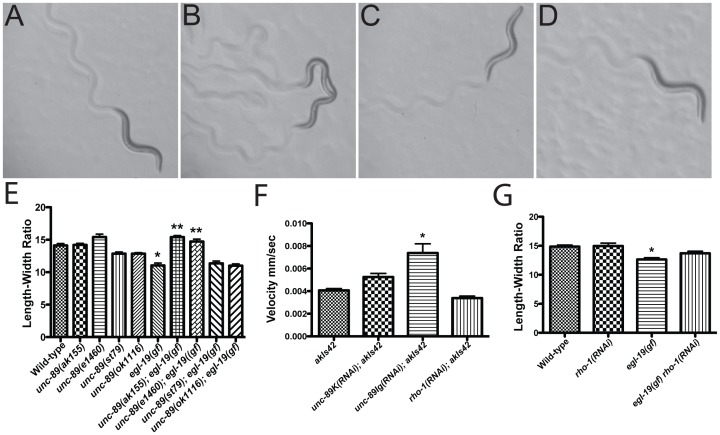
*unc-89* mutations suppress elevated calcium signaling in body wall muscle. (A–D) Representative animals crawling on agar plates. (A) Wild-type animal displaying normal sinusoidal locomotion. (B) Two *vav-1* over expression transgenic animals *(akIs42)* displaying uncoordinated locomotion. (C) *egl-19(tak5)* suppressor mutation in the *vav-1* over expression background displaying normal sinusoidal locomotion. (D) *unc-89(ak155)* suppressor in the *vav-1* over expression background displaying normal sinusoidal locomotion. (E) *egl-19(gf)* is hyper-contracted compared to wild-type and *unc-89* mutants (*p<0.01). The hyper-contraction of *egl-19(gf)* is suppressed in the *unc-89(ak155)* and *unc-89(e1460)* mutant backgrounds (**p<0.01) but not in the *unc-89(st79)* or *unc-89(ok1116)* mutant background (p = 0.48). n = 10 for each genotype. (F) RNAi knockdown of the Ig domain-rich domain isoforms of UNC-89 suppresses the slow uncoordinated locomotion of animals over-expressing *vav-1(akIs42)* (*p<0.01); however, RNAi knockdown of the kinase domain containing isoforms of UNC-89 or *rho-1* does not suppress the slow uncoordinated locomotion of animals over-expressing *vav-1*. (G) *egl-19(gf)* mutants grown on empty vector RNAi are significantly hyper-contracted compare to wild-type animals (*p<0.01). *rho-1(RNAi)* treatment did not significantly suppress the hyper-contraction of *egl-19(gf)* (N.S. p = 0.06). n = 10 for each genotype.

To identify genes involved in the regulation of calcium in muscle cells, we developed a novel mutagenesis screen in the genetically amenable model organism *C. elegans*. Over-expression of *vav-1* (accession number Q45FX5), a Rac GTPase family guanine nucleotide exchange factor, in the body wall muscle leads to erratic calcium signaling and uncoordinated locomotion [18]. Wild-type animals crawl in a rhythmic sinusoidal pattern (Fig. 1A and Video S1); however, when *vav-1* is over-expressed in body wall muscle cells, the animals display a disrupted sinusoidal pattern and slow rate of locomotion (Fig. 1B and Video S2). Thus, we reasoned that a forward genetic screen should identify mutations that can reduce this uncoordinated locomotion. Using this approach, we identified a reduction of function allele of *egl-19(tak5)* and a loss of function allele of *unc-89(ak155)* (Fig. 1A–D and Video S3, S4).

The isolation of a mutation in *egl-19* (accession number Q18698), which encodes an L-type voltage gated calcium channel (L-VGCC) [19] is consistent with VAV-1 regulating intracellular calcium signaling, and indicates that our screen can identify genes involved in calcium regulation. However, the isolation of a mutant allele of *unc-89* (accession number O01761) was not anticipated. Additionally, we have found that a previously isolated reduction of function allele of *egl-19*, *n582* [19], and the canonical allele of *unc-89*, *e1460* [14], [15], can also suppress *vav-1* over-expression (data not shown).

Previous studies have indicated that UNC-89 and mammalian obscurin are important for myofilament assembly and organization in striated muscle [4]–[8], [14]–[17]. However, neither UNC-89 nor obscurin has been implicated in calcium regulation. To investigate whether UNC-89 has a role in calcium signaling, we utilized a gain of function mutation in the L-VGCC, *egl-19(ad695gf)* that has been shown to produce a persistent influx of calcium into body wall muscle cells [19]–[21]. As a result, *egl-19(gf)* mutant animals display a short hyper-contracted body phenotype as compared to wild-type animals (Fig. 1E and Fig. S1). Thus, if UNC-89 amplifies calcium signaling in the body wall muscle, we would anticipate that the introduction of *unc-89(e1460)* or *unc-89(ak155)* mutations into *egl-19(gf)* mutants would suppress the short hyper-contracted body phenotype associated with the increased calcium signaling in these mutants. Consistent with the suppression of *vav-1* over-expression, we found that introduction of *unc-89(ak155)* or *unc-89(e1460)* mutations into *egl-19(gf)* mutants can suppress the hyper-contracted short body phenotype of *egl-19(gf)* mutants (Fig. 1E and Fig. S1). These data suggest that UNC-89 plays a role in calcium mediated muscle contraction.

### Large Ig containing isoforms of UNC-89 are required for optimal calcium signaling in body wall muscle

**Figure 2:**
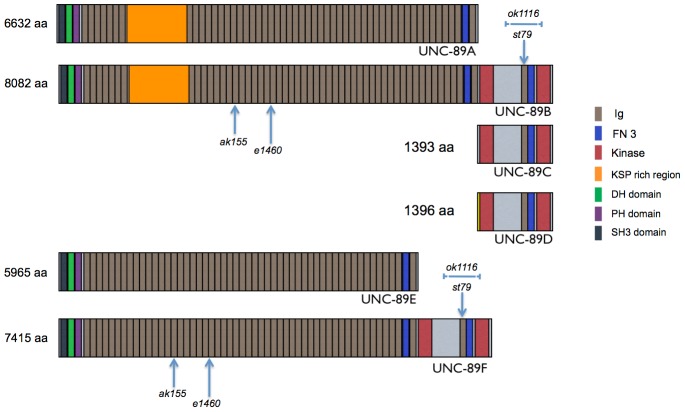
Comparison of UNC-89 isoforms. Schematic of UNC-89 isoforms. The arrows indicate the location of the *unc-89(ak155), unc-89(e1460)* and *unc-89(st79)* stop codons. The blue line underlying *ok1116* indicates the deleted region in *unc-89(ok1116)* mutants.

*unc-89* encodes at least 6 isoforms that utilize different promoter elements, alternative splicing and 3′ untranslated regions to produce UNC-89A, UNC-89B, UNC-89C, UNC-89D, UNC-89E and UNC-89F (Fig. 2) [16], [17]. To determine which isoform(s) is disrupted in *unc-89(ak155)* and *unc-89(e1460)* mutants, we sequenced the *unc-89* genomic region in these animals. We identified nonsense mutations in both *unc-89(ak155)* and *unc-89(e1460)* mutants that introduce stop codons in UNC-89 at Ig repeat 15 and 21, respectively (Fig. 2 and Table S1), which would disrupt the large isoforms (UNC-89A, UNC-89B, UNC-89E, and UNC-89F). The short isoforms, UNC-89C and UNC-89D, are predicted to be intact. This suggests that the large UNC-89 isoforms are important for the regulation of calcium signaling. To investigate whether deletion of the kinase containing isoforms (UNC89B, UNC-89C, UNC-89D and UNC-89F) can suppress *egl-19(gf)*, we utilized *unc-89(st79)*, which contains a stop codon in the Ig domain that lies just N-terminal of the second protein kinase domain (Fig. 2) [17] and *unc-89(ok1116)*, which contains a deletion of the second kinase domain (Fig. 2) [17]. Importantly, these mutants no longer express UNC-89C and UNC-89D small kinase containing isoforms and *unc-89(st79)* no longer expresses the large UNC-89B and UNC-89F isoforms [17]. When either mutation is introduced into the *egl-19(gf)* background, neither can suppress the hyper-contracted short phenotype of *egl-19(gf)* (Fig. 1E), suggesting that the isoforms containing the kinase domains do not promote calcium mobilization in the body wall muscle. Consistent with this result, RNA interference (RNAi) of the kinase containing isoforms of *unc-89* could not suppress the uncoordinated phenotype of *vav-1* over-expression (Fig. 1F), while RNAi of the large UNC-89 isoforms could suppress *vav-1* over-expression, confirming our genetic interaction (Fig. 1F).

In addition to containing several Ig repeats, the large isoforms of UNC-89 contain a Dbl homology domain, which is not found in the short isoforms (Fig. 2). The UNC-89 Dbl homology domain acts as RHO-1/RhoA (accession number Q22038) guanine nucleotide exchange factor [22]. Similarly, the Dbl homology domains from mammalian and zebrafish obscurins have been implicated in RhoA activation [23], [24] as well as the Rho family member TC10 [25]. Since the *C. elegans* genome does not encode a TC10 homolog and the closest relative to TC10 in *C. elegans*, CDC-42, does not interact with the Dbl homology domain of UNC-89 [22], we investigated whether RHO-1 activation through the Dbl homology domain of UNC-89 might be promoting calcium signaling. To accomplish this, we used RNAi knockdown of *rho-1* in an attempt to suppress *egl-19(gf)* or over-expression of *vav-1*. *rho-1(RNAi)* treatment failed to suppress the *egl-19(gf)* short hyper-contracted body phenotype (Fig. 1G) and the uncoordinated locomotion associated with over-expression of *vav-1* (Fig. 1F). Thus, these data suggest that neither the kinase domain-containing UNC-89 isoforms nor the Dbl homology domain are required for promoting optimal calcium signaling. These data demonstrate the importance and specificity of the large Ig repeat rich containing isoforms of UNC-89 in mediating calcium signaling.

### UNC-89 is required for myosin filament organization

Previous studies on *unc-89* mutants and obscurin disruption have demonstrated the importance of UNC-89 and obscurin in myofilament organization and/or assembly [4], [6], [7], [14]–[16], [22]. However, a recent study characterizing the genetic knock out of mouse obscurin has found that obscurin is not important for myofilament assembly [9]. While these findings on obscurin knock out mice are in disagreement with previous studies of obscurin and *unc-89* function, the authors demonstrated that the tandem kinase domain specific isoform of obscurin (KIAA1639) is expressed and Obsl1, an Ig domain-rich protein, is normally localized to the M-line in the obscurin knock out mice [9]. Since the *C. elegans* genome does not encode an apparent Obsl1 homolog, we examined myofilament assembly and organization in *unc-89* mutants.

**Figure 3:**
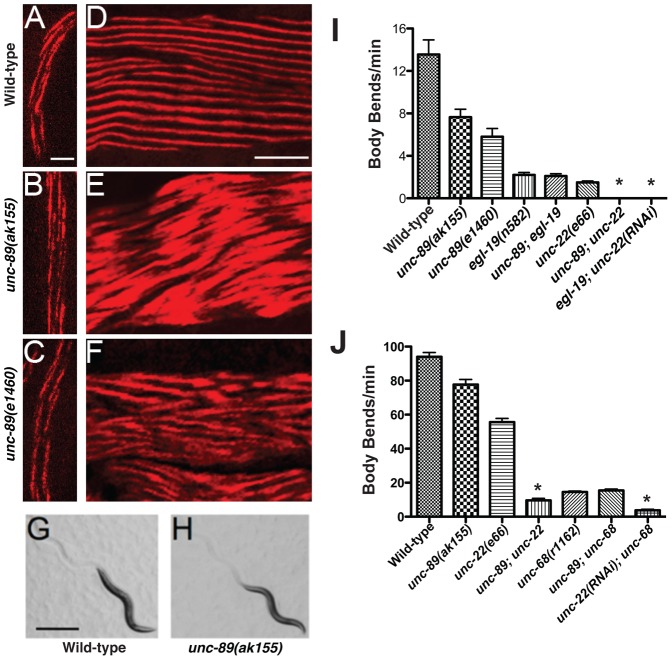
UNC-89 is required for sarcomere organization and function. (A–C) Representative L1 animals labeled with an anti-myosin heavy chain-A (MHC-A) antibody. Scale bar = 2 µm. Anterior is up. (A) Wild-type animal showing normal MHC-A organization. (B, C) *unc-89* mutants indicating normal distribution of MHC-A. (D–F) Representative adult animals labeled with anti-MHC-A antibody. Scale bar = 5 µm. Anterior is to the left. (D) Wild-type animal showing normal MHC-A organization. (E, F) *unc-89* mutants have severely disorganized sarcomeres. Representative (G) wild-type and (H) *unc-89(ak155)* adult animals displaying normal sinusoidal locomotion. Scale bar = 500 µm. (J) Body bend analysis of animals crawling. *egl-19(n582); unc-89(ak155)* display a similar rate of body bends as compared to *egl-19(n582)* single mutants. However, *unc-89(ak155)*; *unc-22(e66)* and *egl-19(n582)unc-22(RNAi)* are completely paralyzed and do not produce any body bends, which is significantly worse than the single mutants (* p<0.05). (K) Body bend analysis of animals swimming. *unc-89(ak155); unc-68(r1162)* mutants show a similar rate of body bends as *unc-68(r1162)* single mutants (p = 0.48). However, *unc-89(ak155); unc-22(e66)* double mutants show a significant decrease in body bends compared to *unc-22(e66)* mutants alone (p<0.001).

Since UNC-89 is expressed during embryogenesis [26], we examined first larval stage animals with anti-myosin immunofluorescence in *unc-89* mutants. Surprisingly, myosin filaments assembled normally in *unc-89(ak155)* and *unc-89(e1460)* larvae and were indistinguishable from wild-type larvae (Fig. 3A–C). This is characterized by myosin positive myofilaments that are organized into A-bands that run along the longitudinal axis of the worm. However, while myosin filaments can assemble in *unc-89(ak155)* and *unc-89(e1460)* mutants, their organization is lost as the mutant animals reach adulthood. In wild-type animals, anti-myosin immunofluorescence labels approximately 10 A-bands per muscle cell that run longitudinally in the antero-posterior axis (Fig. 3D). However, in *unc-89* mutants, myosin is no longer organized into A-bands (Fig. 3E, F). This agrees with a previous report that myosin is poorly organized in adult *unc-89* mutants [22].

As shown in Fig. 3A–F, as the animal develops from larva to adult, the body wall muscle undergoes considerable growth (e.g. two A-bands to >ten A-bands) [27]. Accordingly, our observations are consistent with UNC-89 playing a crucial role in the maintenance of sarcomere organization as the body wall muscle develops. Despite the poor organization of myosin filaments in adult body wall muscle, which is required for locomotion, *unc-89* mutants can crawl fairly normally [14], [17]. Indeed, *unc-89* mutants display relatively normal sinusoidal locomotion on agar plates seeded with a bacterial lawn (Fig. 3G, H). This suggests that myofilament assembly occurs and the filaments are functional in *unc-89* mutants, as indicated by the normal assembly in early larvae and fairly normal locomotion in adults; however, as the *unc-89* mutants develop and grow the organization of the sarcomeres is lost, which indicate that the large Ig domain-rich isoforms of UNC-89 have a critical role in maintaining the organization of the sarcomeres during development. Indeed, transmission electron microscopy analysis of *unc-89(e1460)* mutants indicates clearly formed thick and thin filaments; however, while the filaments are closely aligned, their organization is scattered in the myoplasm [14], indicating UNC-89 is required to maintain sarcomere structure but not filament structure. Furthermore, two recent studies using RNAi knock down of the large isoforms of UNC-89 have implicated UNC-89 in myofibril maintenance [28], [29]. Thus, these data support the notion that during development while muscle is growing, the large Ig rich isoform of UNC-89 is required for maintaining sarcomere organization.

### UNC-89 acts in a common genetic pathway with ryanodine receptors and L-VGCCs to regulate locomotion

To further investigate the role of UNC-89 in muscle activity, we examined body bend frequency as worms crawled on plates or while swimming in liquid. Although *unc-89* mutants exhibit fairly normal sinusoidal locomotion, they displayed a reduced level of body bends compared to wild-type animals, indicating that muscle function is compromised in *unc-89* mutants (Fig. 3I, J). To investigate the role of calcium in muscle activity, we examined mutations in two crucial calcium channels expressed in body wall muscle, the L-VGCC, EGL-19, and the ryanodine receptor (RyR), UNC-68 (accession number Q94279/B7WN66) [19], [30]–[33]. A reduction of function mutation in *egl-19*, leads to a reduced frequency of body bends as compared to wild-type animals (Fig. 3I). To investigate the role of RyRs during locomotion, we examined the rate of body bends of *unc-68* null mutants in liquid since these mutants move negligibly on plates. As anticipated, *unc-68* mutants had a reduced frequency of body bends as compared to wild-type animals (Fig. 3J). Next, we examined genetic interactions between *unc-89* and *egl-19/unc-68* to investigate whether UNC-89 acts in the same genetic pathway as EGL-19 or UNC-68 to regulate muscle contraction. Accordingly, if two proteins act in the same pathway, then the double mutant phenotype should not be worse than the single mutants. However, if the proteins act in separate pathways, the double mutant will have a more severe and pronounced phenotype than the single mutants. Double mutants generated with *unc-89* and *egl-19* or *unc-68* showed a similar phenotype as *egl-19* and *unc-68* single mutants, respectively (Fig. 3I, J). Thus, these data indicate that *unc-89* acts in the same genetic pathway as *egl-19* and *unc-68* to regulate muscle activity. Conversely, when we constructed a double mutant with *unc-89* and *unc-22*, the gene encoding twitchin (accession number Q23551), which is implicated in the regulation of actomyosin interactions during contraction [17], [27], [34], we uncovered a severe phenotypic enhancement of the single mutant phenotype (Fig. 3I, J). In agreement, a similar phenotypic enhancement is observed when *unc-22(RNAi)* is used to knock down UNC-22/twitchin in *egl-19* mutants (Fig. 3I) and *unc-68* mutants (Fig. 3J). Together, these data indicate that UNC-89, UNC-68 RyR and EGL-19 L-VGCC act in a common genetic pathway to regulate muscle contraction that is parallel to the role twitchin plays in muscle contraction. Thus, these results are consistent with UNC-89 playing a critical role in calcium mobilization.

### UNC-89 is required for SR organization

**Figure 4:**
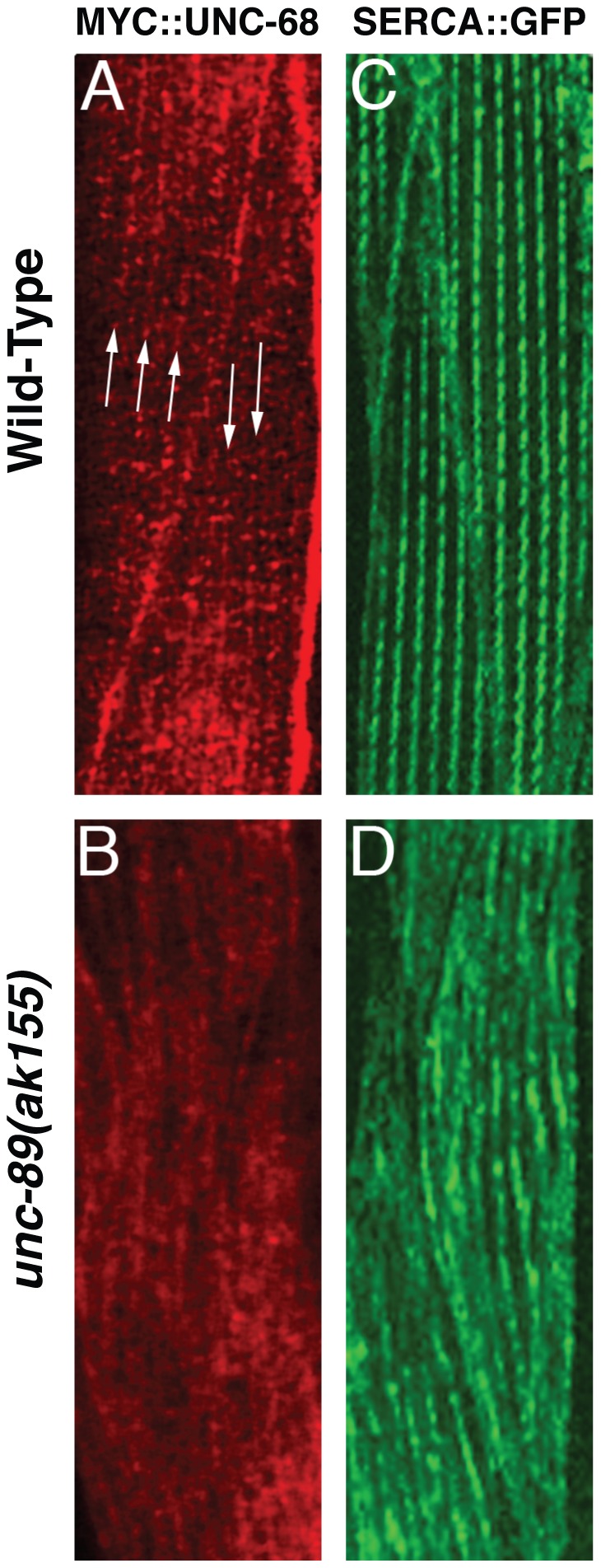
UNC-89 is required for SR protein localization. (A and B) Representative images of RyR (UNC-68) distribution in the body wall muscle of (A) wild-type and (B) *unc-89(ak155)* animals. Arrows in (A) indicate linear punctate pattern of RyRs. (C and D) Representative images of SERCA localization in the body wall muscle of (C) wild-type and (D) *unc-89(ak155)* animals. In *unc-89* mutants (B) RyRs and (D) SERCAs are disorganized. Scale bar = 5 µm.

**Figure 5:**
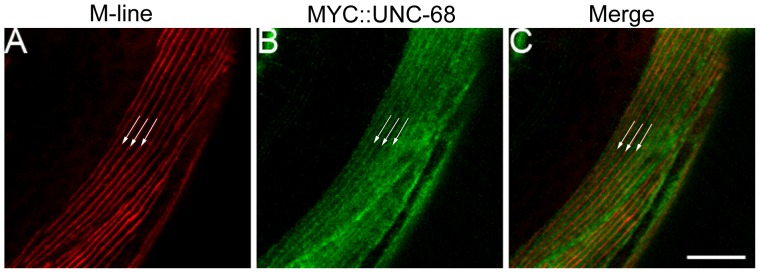
RyRs are organized in a linear pattern that runs parallel and adjacent to the M-line. (A) M-line is labeled with anti-UNC-89 antisera (MH42). (B) MYC::UNC-68 (RyR) labeling. (C) UNC-68 RyRs run parallel and adjacent to the M-line (3 independent M-lines are illustrated by arrows) Scale bar = 10 µm.

Since we have found evidence that UNC-89, RyRs, and L-VGCCs act in a common genetic pathway to regulate muscle contraction and previous studies have implicated mammalian obscurin in regulating the organization of the SR [6], [7], [9], we examined the organization of two SR resident proteins that are involved in SR mediated calcium signaling. First, we investigated the distribution of RyRs, a critical SR membrane calcium channel, in the body wall muscle using myc-tagged RyR [35]. In wild-type animals, RyRs were organized in a linear punctate pattern that runs parallel and adjacent to the M-line (Fig. 4A and Fig. 5). In contrast, the regular distribution and organization of RyRs is lost in *unc-89(ak155)* mutants (Fig. 4B). Second, we examined the localization of the sarco-endoplasmic reticulum calcium ATPase (SERCA) (accession number Q9XTG6), an essential SR calcium reuptake pump, in the body wall muscle. Similar to RyR organization, the distribution of SERCA::GFP in the body wall muscle is localized in a highly organized linear pattern that runs parallel to the M-line in wild-type animals (Fig. 4C) [36]. However, in *unc-89(ak155)* mutants, the distribution of SERCA is disorganized (Fig. 4D). To specifically address whether the large Ig domain-rich isoforms play a role in SERCA distribution, we examined SERCA::GFP expressing animals treated with RNAi specific to the kinase containing isoforms or the large Ig domain-rich isoforms of UNC-89. While treatment with RNAi specific to the kinase containing UNC-89 isoforms resembled control RNAi (Fig. S2A, B), treatment with RNAi specific to the large Ig domain-rich isoforms resulted in a disorganized distribution of SERCA (Fig. S2C). Thus, these results indicate that the large Ig containing isoforms of UNC-89 are important for the localization of RyRs and SERCA in the body wall muscle.

### UNC-89 is required for optimal calcium signaling in the pharyngeal and body wall muscle

**Figure 6:**
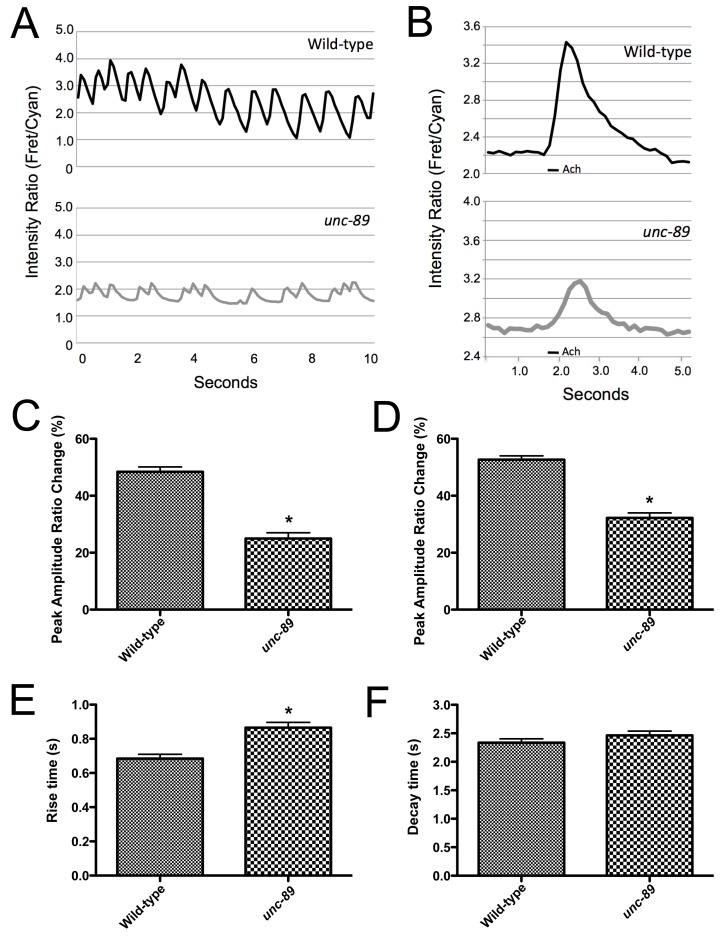
UNC-89 is required for optimal calcium signaling. (A) Representative spontaneous pharyngeal muscle calcium traces from wild-type and *unc-89(ak155)* animals. (B) Representative ACh induced body wall muscle calcium traces from wild-type and *unc-89(ak155)* animals. (C) Quantification of peak amplitude change upon spontaneous pharyngeal muscle contraction revealed a less robust calcium transient in *unc-89(ak155)* mutants as compared to wild-type animals (n = 20)(* p<0.01). (D) Quantification of peak amplitude change upon ACh induced body wall muscle contraction revealed a less robust calcium transient in *unc-89(ak155)* mutants as compared to wild-type animals (n = 20)(* p<0.01). (E) Rise time to peak calcium concentration upon Ach application is delayed in *unc-89(ak155)* mutants (* p<0.01); however, (F) the decay time is not significantly different from the wild-type response.

Since SERCA and RYRs are disorganized in *unc-89* mutants and UNC-89 acts in a common genetic pathway with RyRs and L-VGCCs to regulate muscle contraction, we directly investigated whether calcium release was affected in *unc-89* mutants by measuring calcium transients. Since UNC-89 is expressed in, and is required for, the sarcomeric organization and activity in both pharyngeal and body wall muscle [14]–[17], we examined spontaneous calcium transients in pharyngeal muscle and acetylcholine (ACh) induced calcium transients in body wall muscle, using the genetically encoded calcium sensor, YC3.60 [37] that is provided by an integrated transgene. While analysis of *unc-89(ak155)* mutants revealed reliable calcium transients in both the pharyngeal muscle (Fig. 6A) and body wall muscle (Fig. 6B), the peak amplitude of these calcium transients were reduced compared to wild-type animals (Fig. 6C, D). Furthermore, *unc-89* mutants show a delay in the time required to reach maximal calcium concentration (rise time); however, the time to reach baseline (decay time) was not significantly different from wild-type animals (Fig. 6E, F). These data are consistent with our genetic and behavioral analyses and directly demonstrate that UNC-89 has a crucial role in calcium mobilization in pharyngeal and body wall muscle. Furthermore, the reduction of and delay in reaching peak calcium levels indicates that the large Ig domain-rich UNC-89 isoforms are critical for optimal E-C coupling in *C. elegans* muscle.

## Discussion

E-C coupling is the response in muscle to membrane depolarization and involves calcium entering into the muscle cytoplasm through L-VGCCs, which directly stimulate adjacent RyRs embedded in the SR membrane. The activation of RyRs leads to an increase in cytoplasmic calcium, which triggers muscle contraction. Cytoplasmic calcium is then rapidly removed by the SR resident SERCA pumps as well as other plasma membrane calcium selective pumps and exchangers. We have found that the large Ig domain-rich isoforms of UNC-89 act as a structural scaffold that maintains the sarcomeres and the SR in the appropriate subcellular configuration for optimal calcium mobilization and E-C coupling. Since both SERCAs and RYRs are mislocalized in *unc-89* mutants lacking the large Ig domain-rich isoforms, it is unlikely that UNC-89 directly interacts with these components. Rather, loss of the large Ig domain-rich UNC-89 isoforms leads to overall sarcomere and SR disorganization and the subsequent mislocalization of SERCAs and RYRs in the SR. Additionally, since calcium transients are reduced in *unc-89* mutants and these mutant animals cannot crawl or swim as vigorously as their wild-type counter parts, these results indicate that the spatial positioning of SR components and sarcomeres are critical for accurate and efficient calcium dependent muscle contraction. Two recent studies have found that disruption of cytoplasmic actin and tropomodulin 3 in skeletal muscle leads to SR disorganization, reduced calcium release and muscle function [38], [39]. These results along with our data presented here, illustrate the necessity of structural organization for appropriate calcium signaling in muscle cells and that this organization is crucial for optimal muscle performance.

Since L-VGCCs play a critical role in E-C coupling, we attempted to analyze the localization of EGL-19 L-VGCC in the body wall muscle of *unc-89* mutants. To accomplish this, we utilized a EGL-19::mCherry fusion protein [40] that is capable of rescuing *egl-19* mutants. While we could visualize EGL-19::mCherry localization in the body wall muscle, unfortunately the sub-cellular localization of EGL-19::mCherry was not readily reproducible in wild-type animals. Thus, it was not possible to determine whether the distribution of EGL-19 is perturbed in *unc-89* mutants. Nevertheless, our genetic analysis indicates that UNC-89 acts in a common genetic pathway with EGL-19 L-VGCC and UNC-68 RyR and our calcium imaging data indicates that UNC-89 is critical for optimal calcium signaling the body wall muscle. Thus, these data together reveal a key functional role of UNC-89 in optimizing calcium mediated muscle contraction, which is critical for efficient and effective muscle contraction.

Additionally, we have found that UNC-89, UNC-68 RyR and EGL-19 L-VGCC act in parallel to UNC-22/Twitchin to regulate muscle contraction (Fig. 3I, J). Twitchin is another giant muscle protein, related to titin and obscurin, which is found in invertebrate muscle where it plays a role in negatively regulating the rate of muscle relaxation [17], [34]. Accordingly, twitchin is postulated to promote the tethering of myosin filaments with actin filaments [41]. Consistent with this physical interaction, our genetic analysis indicates that twitchin plays a parallel role to calcium in promoting muscle contraction and suggests that the large Ig domain-rich isoforms of UNC-89 do not have a direct role in actomyosin interactions. However, our present analysis does not rule out a role for the small kinase containing isoforms of UNC-89 (UNC89C and UNC-89D) in actomyosin interactions.

In *C. elegans*, several genes have been identified that play a key role in myofilament organization. Mutations in these genes lead to uncoordinated locomotion, paralysis or embryonic lethality [14], [27]. In contrast, *unc-89* mutants are capable of fairly normal sinusoidal locomotion, yet have severely disorganized myofilaments. Moreover, while the sarcomeres are disorganized in *unc-89* adults animals, in young *unc-89* larvae the myofilaments are indistinguishable from wild-type animals. Furthermore, examination of adult muscle ultrastructure reveals that while the myofilaments were scattered in the myoplasm, they are still closely aligned [14]. This data argues that although the sarcomeres are disorganized in *unc-89* mutants, the sarcomeres are functional; however, due to reduced calcium mobilization muscle activity is attenuated in *unc-89* mutants. Moreover, these data reveal the importance of the large Ig domain-rich isoforms of UNC-89 in maintaining sarcomere and SR organization or adding new sarcomeres as the muscle develops, grows, becomes more complex and is under more force.

While several studies have implicated UNC-89 and obscurin in myofilament assembly and organization [3]–[8], [14]–[17], surprisingly a recent characterization of obscurin-null mice has revealed normal sarcomeric structure [9]. However, the SR in the obscurin-null mice is disorganized [9]. Interestingly, obscurin-null mice still express the small kinase containing isoform of obscurin (KIAA1639) and obsl1, an Ig domain-rich protein, localizes normally in the M-line. This is interesting because in mammals, obscurin is localized to the periphery of the myofibrils where it is thought to link the SR to the sarcomere [42], [43]. Moreover, the relatively small Ig domain-rich obscurin homolog, obsl1, is an integral component of the M-line [44], where it may play a role in M-line organization. Consistent with this premise, several muscular dystrophies that arise from mutations in the obsl1 and obscurin binding domain of titin result in the mislocalization of obsl1 and lead to the gradual failure of sarcomere maintenance [44]. In *C. elegans*, which do not possess an apparent obsl1 homolog, UNC-89 localizes to the M-line [14]–[17]. Furthermore, in *unc-89* mutants, components of the SR are disorganized and, while sarcomere assembly is normal in early development, maintenance of the sarcomeres is lost in adults. Thus, our data indicates that UNC-89 has two roles: maintenance of M-line integrity, like obsl1, and organization of the SR, like peripheral obscurin. In light of our data, it will be informative to investigate the loss of both obscurin and obsl1 in mice to reveal the level of compensation that may occur in the absence of each other and whether loss of both obscurin and obsl1 display similar muscle defects as observed in *unc-89* mutants.

## Materials and Methods

### Nematode strains, maintenance and suppressor isolation

Nematodes were grown at 20C and the *E. coli* strain OP50 was used as a food source. Wild-type nematodes were the N2 strain. Strains used for this study are as follows: *unc-89(ak155)* I, *unc-89(e1460)* I, *unc-89(st79)* I, *unc-89(ok1116)* I, *egl-19(tak5)* IV, *egl-19(n582)* IV, *egl-19(ad695)* IV, *unc-22(e66)* IV, *rho-1(ok2418)* IV, *unc-68(r1162)* V, *akIs42 [Pmyo-3::vav-1]*, *akIs104 (Pmyo-3::YC3.60)*, *akIs106 (Pmyo-2::YC3.60)*, *sca-1::GFP* [32], *cimEx4(egl-19::mCherry)* [40], *zwIs108* (*Pmyo-3::MYC::unc-68)* [31].

In two separate mutagenesis screens, a strain overexpressing *vav-1* in the body wall muscle (*akIs41*) was mutagenized with 50 mM ethylmethane sulfonate using standard procedures [45]. Approximately 12,500 haploid genomes were screened for animals with improved locomotion. From these genetic screens, we identified 11 suppressor mutants, two of which are *egl-19(tak5)* and *unc-89(ak155)*. These mutant loci were mapped to their chromosomal position by single nucleotide polymorphism mapping using standard procedures [46], [47]. The genes affected by these mutant alleles were identified by genetic complementation and genomic sequencing (*ak155*)(details below) or rescue of mutant phenotype (*tak5*) with *cimEx4(egl-19::mCherry)* [40].

### Determination of mutation sites in unc-89(ak155) and unc-89(e1460)

Genomic DNA from each strain was prepared and shotgun libraries were prepared following instructions from Illumina. The entire genomic sequence from each strain was determined by sequencing shotgun clones on an Illumina HiSeq with 100 base reads and the program SCS2.8 was used to process the run and call the bases. The sequence files were imported into CLC Genomics Workbench 4.8 and trimmed to </ = 1 ambiguous bases with a minimum read length of 36 bases. The trimmed reads were mapped against the *C. elegans* genomic sequence obtained from WormBase.org (Oct. 2011), using stringent parameters (0.7 on the length and 0.8 similarity), yielding an average coverage of >50X. SNPs and InDels were identified with minimum coverage requirements of >25X. Our analysis was then focused on the ∼60 kb *unc-89* gene. For each mutant strain, a single homozygous mutation in the *unc-89* coding sequence was identified. Each of these candidate mutations was verified by using the Sanger method to sequence ∼600 bp segments PCR-amplified from single worms of each strain.

### DNA constructs and transgenesis

Pmyo-3::YC3.60 construct was assembled by PCR amplifying YC3.60 (kind gift from Atsushi Miyawaki) with primers containing NheI and Acc65I restriction sites. The PCR amplified YC3.60 was digested with NheI and Acc65I and cloned into the vector containing the *myo-3* promoter vector, pPD95.86 (Addgene). Similarly, Pmyo-2::YC3.60 construct was assembled by PCR amplifying YC3.60 with primers containing NheI and Acc65I restriction sites. The PCR amplified YC3.60 was digested with NheI and Acc65I and cloned into the vector containing the *myo-2* promoter vector, pPD94.48 (Addgene). Using standard procedures [48], these constructs along with pJM23 [*lin-15(+)*] were injected into *lin-15(n765ts)* animals. Transgenic progeny were identified by body wall or pharyngeal muscle fluorescence and *lin-15* mutant rescue. Stable integrated lines were generated with X-rays using standard procedures [48].

### Body hyper-contraction analysis

Images were taken on a Zeiss Discovery V12 dissecting microscope with a 3.5× PlanApo S objective using a Canon Powershot G11 digital camera of young adult animals (10 animals were imaged for each genotype). Images were processed and analyzed using ImageJ. Body size was determined by length (µm, tip of nose to tip of the tail) divided by width (µm, width at the vulva). Additionally, tip of the nose to the posterior edge of the pharyngeal terminal bulb (mm) was measured to investigate hyper-contraction of the body.

### RNA interference

L4 animals were grown and allowed to feed on *E. coli* [HT115 (DE3)] containing an RNAi construct specific to the kinase containing isoforms of UNC-89 (JA:C24G7.5) [49], the large Ig containing isoforms of UNC-89 (JA:C09D1.1) [50], UNC-22/Twitchin (pLT61.1) [51] or *rho-1* [18]. Phenotypic analysis was carried out on young adult first generation progeny. For the *rho-1(RNAi)* experiments, since exposure of L4 animals for 24–36 hours to *rho-1(RNAi)* results in ∼100% embryonic lethality in the F1 progeny, L1 larvae were grown and allowed to feed on bacteria carrying an RNAi construct specific *rho-1* for approximately 48 hours [18], [52]. Phenotypic analysis of *rho-1(RNAi)* treated animals was carried out once these animals developed into young adults. RNAi knockdown was verified by observing reported phenotypes in RNAi treated animals [14]–[18], [53]. Empty vector, pPD129.36 (Addgene), was used as a control.

### Immunohistochemistry and Microscopy

Animals were fixed in Bouin's fixative for one hour at room temperature. After the initial 30 minutes the animals were freeze cracked by incubation in an ethanol dry ice bath for two minutes and rapidly thawed in hot tap water. Fixed animals were thoroughly washed in borate buffer (20 mM boric acid, 10 mM NaOH, pH 9.5, containing 0.5% Triton X-100, 2% Beta-mercaptoethanol). Antibodies were added in standard phosphate buffer saline containing 0.5% Triton. Antibodies used were a myosin heavy chain A antibody, DM5.6, (Developmental Studies Hybridoma Bank) [54], UNC-89 antibody to label the M-line (MH42) [15] and Myc antibody (Cell Signaling Technology). UNC-68 and SERCA were localized using a Myc tagged UNC-68 [35] and SERCA::GFP fusion protein [36]. Fixed and labeled specimens were analyzed and imaged on a Zeiss LSM 510 laser scanning confocal microscope. Images were processed in Photoshop (Adobe) and arranged in Illustrator (Adobe).

For calcium imaging in the pharynx, animals expressing *Pmyo-2::YC3.60* were immobilized in M9 containing 1 mM levamisole and 10 mM 5-hydroxytryptamine. For calcium imaging in the body wall muscle, animals expressing *Pmyo-3::YC3.60* were immobilized using cyanoacrylate surgical glue (3M) and dissected on a polydimethylsiloxane (Sylgard) coated cover slip in extracellular fluid [150 mM NaCl, 5 mM KCl, 4 mM MgCl_2_, 1 mM CaCl_2_, 15 mM HEPES, and 10 mM glucose, (pH 7.4, 340 mOsm)]. The intestine and gonad are removed by mouth suction pipette and the basement membrane is removed by collagenase and proteinase K treatment for 30 seconds. To apply ACh (100 µM), a pipette was placed close to the body wall muscle and applied by pressure ejection to deliver a short duration of ACh (∼100 millisecond). Images were collected on a Zeiss AxioObserver equipped with an optical beam splitter (Photometrics) and a Hamamatsu ORCA-R2 charged-coupled device camera using Zeiss AxioVision software. Image analysis was carried out in AxioVision using the physiology module and exported to Excel (Microsoft) for presentation and Prism (GraphPad) for statistical analysis.

### Behavioral analyses

L4 animals of each genotype were picked to fresh OP50 plates and allowed to mature at 20°C for 24 hours. The swimming assays were carried out on individual young adult animals in M9 by counting complete body deflections for 30 seconds. For locomotion (crawling), body bends of individual young adult animals were counted for 1 minute. A body bend was scored as a complete deflection of the anterior portion of the nematode from the midline. 20 animals of each genotype were analyzed. Animal velocity was determined by video microscopy tracking of multiple worms using an Olympus SZ61 stereomicroscope equipped with an Sony SXD-900 charge-coupled device camera. Movies were taken and Velocity was calculated using MatLab (Mathworks) software [55].

### Statistical analysis

Statistical Analyses were carried out in Excel (Microsoft) or GraphPad (Prism). Data was presented as means ± SEM. Statistical significance was determined using a Student's two tailed t test or ANOVA followed by Dunn's multiple comparison test. P values<0.05 were taken to indicate statistical significance.

## Supporting Information

Figure S1 **The large Ig domain-rich isoforms of UNC-89 are required for the** ***egl-19(gf)*** **induced hyper-contracted body phenotype.** Tip of the nose to the posterior edge of the pharynx measurements were determined. *egl-19(ad695gf)* animals were hyper-contracted compared to wild-type animals (* p<0.05). Introduction of either *unc-89(ak155)* or *unc-89(e1460)* into the *egl-19(ad695gf)* background significantly reduced the hyper-contracted body phenotype (**p<0.05), whereas *unc-89(st79)* and *unc-89(ok1116)* had no affect on the *egl-19(ad695gf)* hyper-contracted body phenotype. n = 10 for each genotype.
(TIF)

Figure S2 **The large Ig rich isoforms of UNC-89 are required for normal localization of SERCA.** (A) Representative image of SERCA::GFP localization in a wild-type animal. (B) Representative image of SERCA::GFP localization in a wild-type animals treated with RNAi specific to the kinase containing isoforms of UNC-89. (C) Representative image of SERCA::GFP localization in a wild-type animals treated with RNAi specific to the large Ig domain-rich isoforms. Note the disorganization of the linear punctate structures in the wild-type animals treated with the RNAi specific to the large Ig domain-rich isoforms of UNC-89. Scale bar = 5 µm.
(TIF)

Table S1 **Shows the sequence alteration in the** ***unc-89*** **coding region in** ***unc-89(ak155)*** **and** ***unc-89(e1460)*** **mutants.**
(DOCX)

Video S1 **Shows wild-type locomotion.**
(MOV)

Video S2 **Shows uncoordinated locomotion of animals over-expressing** ***vav-1*** **.**
(MOV)

Video S3 **Shows wild-type like locomotion of** ***egl-19(tak5)*** **mutant animals over- expressing** ***vav-1*** **.**
(MOV)

Video S4 **Shows wild-type like locomotion of** ***unc-89(ak155)*** **mutant animals over- expressing** ***vav-1*** **.**
(MOV)
