# Supplementary figures and images for: Large Isoforms of UNC-89 (Obscurin) Are Required for Muscle Cell Architecture and Optimal Calcium Release in Caenorhabditis elegans
Source: PLoS One. 2012 Jul 2;7(7):e40182. doi: 10.1371/journal.pone.0040182 (PMC3388081; doi:10.1371/journal.pone.0040182)

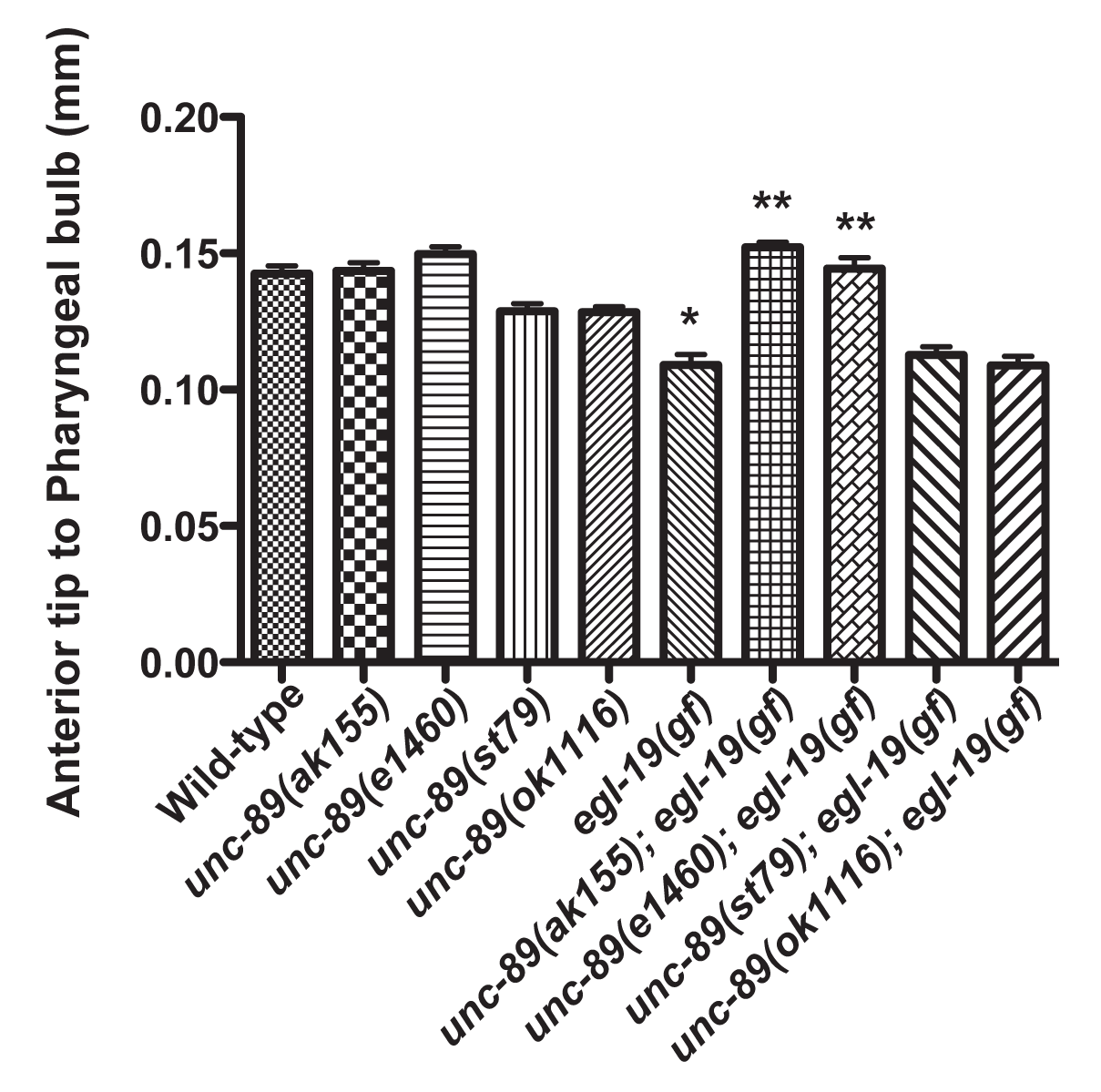

Supplement: Figure S1 — The large Ig domain-rich isoforms of UNC-89 are required for the egl-19(gf) induced hyper-contracted body phenotype. Tip of the nose to the posterior edge of the pharynx measurements were determined. egl-19(ad695gf) animals were hyper-contracted compared to wild-type animals (* p<0.05). Introduction of either unc-89(ak155) or unc-89(e1460) into the egl-19(ad695gf) background significantly reduced the hyper-contracted body phenotype (**p<0.05), whereas unc-89(st79) and unc-89(ok1116) had no affect on the egl-19(ad695gf) hyper-contracted body phenotype. n = 10 for each genotype. (TIF) [file pone.0040182.s001.tif]

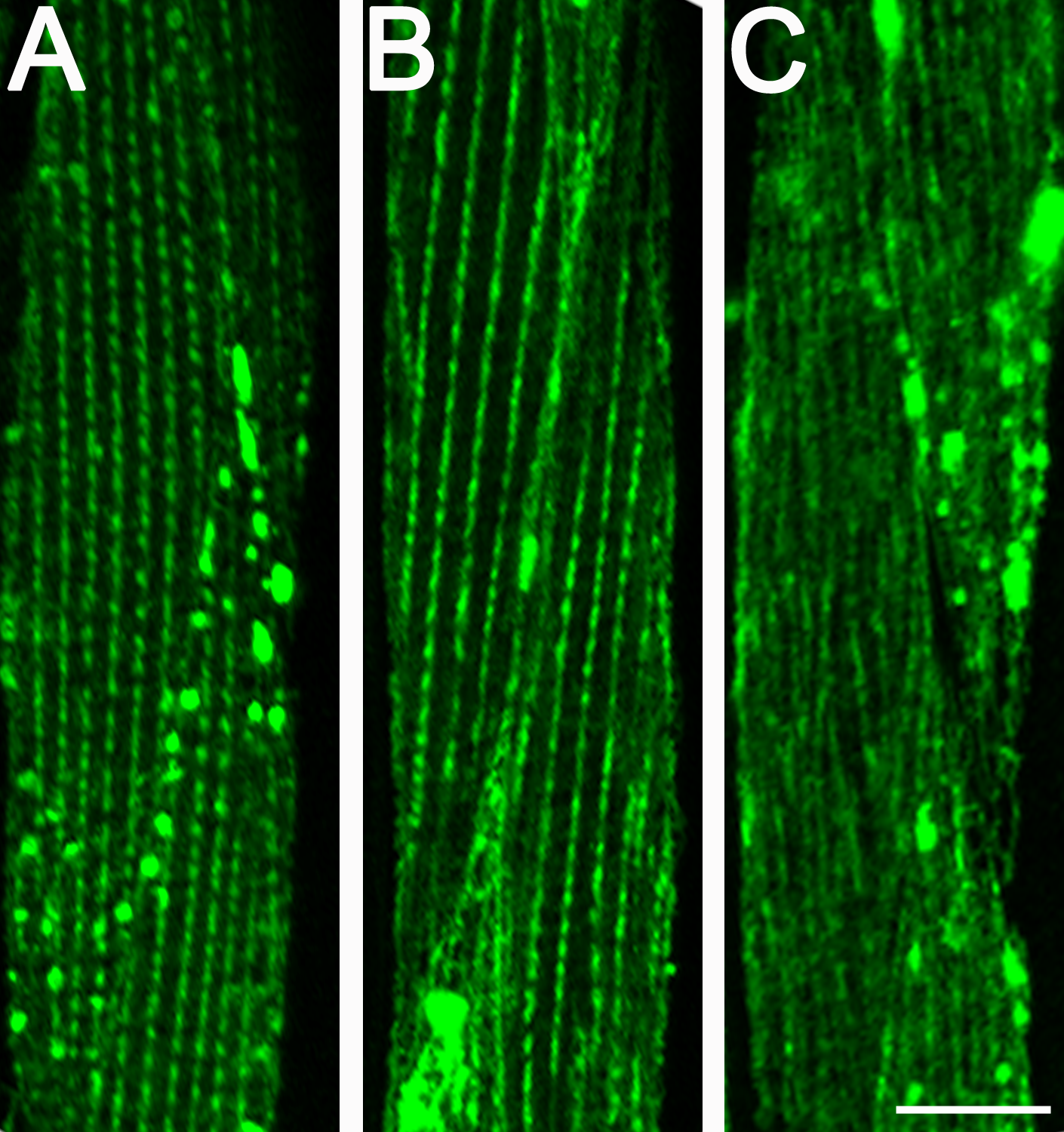

Supplement: Figure S2 — The large Ig rich isoforms of UNC-89 are required for normal localization of SERCA. (A) Representative image of SERCA::GFP localization in a wild-type animal. (B) Representative image of SERCA::GFP localization in a wild-type animals treated with RNAi specific to the kinase containing isoforms of UNC-89. (C) Representative image of SERCA::GFP localization in a wild-type animals treated with RNAi specific to the large Ig domain-rich isoforms. Note the disorganization of the linear punctate structures in the wild-type animals treated with the RNAi specific to the large Ig domain-rich isoforms of UNC-89. Scale bar = 5 µm. (TIF) [file pone.0040182.s002.tif]
