# Supplementary material for: Large Isoforms of UNC-89 (Obscurin) Are Required for Muscle Cell Architecture and Optimal Calcium Release in Caenorhabditis elegans
Source: PLoS One. 2012 Jul 2;7(7):e40182. doi: 10.1371/journal.pone.0040182 (PMC3388081; doi:10.1371/journal.pone.0040182)
Supplement: Table S1 — Shows the sequence alteration in the unc-89 coding region in unc-89(ak155) and unc-89(e1460) mutants. (DOCX) [file pone.0040182.s003.docx]

Table S1. Molecular lesions in *unc-89* mutants

| **Allele** | **Nucleotide change in *unc-89b**** | **Description of Molecular lesion in UNC-89B*** |
| --- | --- | --- |
| *unc-89(ak155)* | C to T change at position 7801 | Q2601Stop |
| *unc-89(e1460* | C to T change at position 9577 | Q3193Stop |

* *unc-89b* DNA coding sequence and UNC-89B amino acid sequence were obtained from wormbase.org
